# Anxiety and panic disorder: First-line pharmacotherapy

**DOI:** 10.6026/973206300223425

**Published:** 2026-06-30

**Authors:** Shruti Vihang Brahmbhatt, Akil Idrish Sunasara, Mahavirsingh Rajput

**Affiliations:** 1 Department of Pharmacology, Shrimati Bhikhiben Kanjibhai Shah Medical Institute and Research Centre, Sumandeep Vidyapeeth Deemed to be University, Piparia, Vadodara- 391760, Gujarat, India

**Keywords:** Anxiety disorder, panic disorder (PD), first-line pharmacotherapy, selective serotonin reuptake inhibitors (SSRIs), benzodiazepines

## Abstract

Anxiety and panic disorders are common psychiatric illnesses, but real-world data on first-line pharmacotherapy outcomes from Indian tertiary care settings remain limited. Therefore, it is of interest to evaluate prescribing patterns, efficacy, safety, and tolerability of first-line pharmacotherapy in 40 newly diagnosed patients with anxiety or panic disorder. Anxiety disorder accounted for 72.5% of cases, and SSRIs alone or combined with benzodiazepines were the most frequently prescribed treatment regimens. Favorable clinical outcomes were observed in 77.5% of patients, while adverse drug reactions were predominantly mild and did not require treatment discontinuation. Thus, SSRI-based first-line pharmacotherapy demonstrated good efficacy and tolerability, supporting guideline-based management of anxiety and panic disorders.

## Background:

According to the Global Burden of Disease (GBD) study, anxiety disorders affected nearly 359 million people globally in 2021 and significantly impaired quality of life, social functioning and work productivity [1]. In India, anxiety disorders remain highly prevalent with a significant treatment gap, especially among young adults and urban populations [2]. Anxiety and panic disorders are often associated with depression and substance use disorders, making early diagnosis and treatment essential to prevent chronicity and relapse [3]. Anxiety disorders are characterized by excessive fear, worry, restlessness, irritability and sleep disturbances causing functional impairment [4]. Panic disorder is defined by recurrent unexpected panic attacks associated with palpitations, dizziness, trembling and fear of losing control, which may lead to significant functional impairment if untreated [5, 6]. Although cognitive behavioural therapy is effective, SSRIs and SNRIs remain the preferred first-line pharmacotherapy for anxiety and panic disorders because of their efficacy and safety profile [7, 8, 9, 10, 11]. Benzodiazepines may provide short-term symptomatic relief but require cautious use because of dependence and withdrawal risks [10, 12]. Despite guideline recommendations, benzodiazepines such as alprazolam and clonazepam are frequently overprescribed in Indian clinical practice, raising concerns regarding long-term safety and irrational prescribing [13]. Therefore, the ICMR has emphasized evidence-based and judicious use of psychotropic medications in routine practice [14]. Therefore, it is of interest to report the prescribing pattern and clinical outcomes of first-line pharmacotherapy in newly diagnosed patients with anxiety and panic disorders at a tertiary care teaching hospital.

## Materials and Methodology:

## Study design:

[1] Type of study - A prospective observational study.

[2] Source of data - Psychiatry Department of a Tertiary care teaching hospital

[3] Sample description - Patients were recruited using consecutive sampling during the study period.

This observational (non-interventional) study was conducted at a tertiary care teaching hospital after Institutional Ethics Committee approval. 40 patients with Anxiety and Panic Disorder were enrolled with written informed consent. Data were collected from case files and recorded in case record forms. Adverse drug reactions were documented and reported to the institutional ADR Monitoring Centre for analysis.

## Patient selection criteria:

## Inclusion criteria:

[1] Patients' diagnosed first time with Anxiety or panic disorder according to DSM-5 criteria are included in the study.

[2] Patients aged between 18 and 65 years are included in the study.

[3] Patients who are able to provide informed consent, or whose legal guardian provides consent when applicable, are included in the study.

## Exclusion criteria:

[1] Diagnoses of any other psychiatric disorder other than anxiety or panic disorder were excluded in the study.

[2] Pregnant or breastfeeding women are not included in the study.

[3] Studies involving paediatric or adolescent populations (<18 years) are excluded.

[4] Patient or legal guardians who are unwilling to provide informed consent are excluded.

## Methodology:

The proposed study is prospective observational in nature.

## Sample size calculation:

The formula to calculate sample size:

n=4pq/(L^2^)

Where:

n = required sample size

p = estimated prevalence

l = margin of error (precision)

q= Complement of the prevalence (p).

This is the probability of the event not occurring.

Mathematically,

q = 100 - p

With respect to reference DeGeorge *et al.* [12] prevalence=8%

As the prevalence of GAD available we have P = 8% and calculated sample size as follows

p = 8 %

q = 100-8 = 92 %

Taking, L (margin of error) = 10%

n=(4*(8)*(92))/(4)^2^

n = 29

To enhance the precision of the study, a sample size of 40 has been selected. The study sample will consist of 40 patients diagnosed with anxiety and panic disorder who visit the psychiatry department during the study period. Although the calculated minimum sample size was 29, a total of 40 patients were ultimately included to enhance precision within the study duration.

## Statistical methods:

This was a Questionnaire based observational survey study. A suitable data collection form i.e.; case record form was prepared and used to collect the data. Gathered data was entered in Microsoft excel and its percentage analysis was done.

## Results:

The mean age was 36.3 ± 10.8 years (range: 18-57), with most patients aged 31-45 years (42.5%), followed by ≤30 years (30.0%) and >45 years (27.5%). Males comprised 52.5% and females 47.5%, with a mean body weight of 65.7 ± 12.8 kg (range: 45-92 kg). Anxiety disorder was diagnosed in 72.5% and panic disorder in 27.5% of patients. Most patients (67.5%) had no comorbidities; among those who did, hypertension (20.0%) and diabetes mellitus (12.5%) were most common. The socio-demographic and clinical characteristics of the study population are summarized in this Table 1. Of 40 newly diagnosed patients, anxiety disorder was the most common diagnosis (72.5%, n = 29), followed by panic disorder (27.5%, n = 11) based on DSM-5 criteria (Figure 1 - see PDF). Combination therapy was common; sertraline + clonazepam were prescribed in 17.5% of patients and desvenlafaxine + clonazepam in 15%. Among antidepressant monotherapy, escitalopram was most frequent (12.5%), followed by duloxetine and fluoxetine (5% each) (Figure 2 - see PDF). Most patients (26/40) reported no adverse drug reactions. Among those who did, dizziness was most common, followed by nausea and sedation. All reactions were mild and required no treatment discontinuation, indicating good tolerability (Figure 3 - see PDF). Clinically, 14 patients achieved complete recovery and 17 were recovering, while 9 showed inadequate improvement, reflecting an overall favorable treatment response (Figure 4 - see PDF). Patients who recovered were predominantly treated with SSRIs, as monotherapy or combined with benzodiazepines, providing sustained symptom control, while benzodiazepines aided early relief during initial treatment. Non-recovered patients likely had higher symptom severity or shorter treatment duration. Overall, first-line pharmacotherapy yielded favorable outcomes, with benzodiazepines facilitating early clinical stabilization.

## Discussion:

The present prospective observational study was conducted to evaluate the prescribing pattern and clinical outcomes of first-line pharmacotherapy in patients newly diagnosed with anxiety disorder and panic disorder at a tertiary care teaching hospital. Anxiety disorder constituted the majority of cases, accounting for 72% (n = 29) of the study population, while panic disorder was identified in 28% (n = 11) of patients. According to Gautam *et al.* similar observations have been reported in Indian hospital-based studies, where generalized anxiety disorders were more commonly diagnosed at first clinical contact compared to panic disorder [15]. The episodic and unpredictable nature of panic attacks may contribute to delay help-seeking, explaining the relatively lower proportion of panic disorder cases. The socio-demographic analysis revealed a mean age of 36.3 ± 10.8 years, with 42.5% of patients belonging to the 31-45 years age group, indicating that these disorders predominantly affect individuals during their economically productive years. According to Math and Srinivasaraju Indian epidemiological studies have consistently reported that psychological stress related to occupation, financial responsibilities and family obligations predisposes individuals in this age group to anxiety-related disorders [16]. The near-equal gender distribution (52.5% males and 47.5% females) reflects increasing mental health awareness among both genders, consistent with recent Indian hospital-based studies demonstrating a narrowing gender gap [2]. Regarding pharmacological management, benzodiazepines such as alprazolam and clonazepam were the most frequently prescribed drugs, each constituting 20% of prescriptions, attributed to their rapid onset and effectiveness in acute symptom relief. Similar prescribing trends have been reported in Indian drug utilization studies in tertiary care settings [13]. However, benzodiazepines were primarily used as short-term agents or adjuncts to antidepressant therapy, reflecting cautious prescribing in view of their risk of dependence and withdrawal. Combination therapy was frequently utilized. Sertraline and clonazepam were prescribed in 17.5% of patients, while desvenlafaxine and clonazepam were used in 15% of cases. According to Shilpa *et al.* this combination approach has been reported in Indian tertiary care studies and is considered practical in patients with moderate to severe anxiety symptoms [17], balancing early symptom control with long-term disease management. Among antidepressants, SSRIs constituted the backbone of pharmacotherapy. Escitalopram was the most frequently prescribed SSRI as monotherapy (12.5%), followed by sertraline and fluoxetine. Current Indian and international guidelines recommend SSRIs as first-line agents for both anxiety and panic disorder [18]. SNRIs such as desvenlafaxine and duloxetine were prescribed in patients with more severe symptoms or partial response to SSRIs, reflecting individualized treatment decisions. In the present study, 67.5% of patients had no associated medical comorbidities. However, hypertension (20%) and diabetes mellitus (12.5%) were the most frequently observed comorbid conditions. Indian studies have demonstrated a bidirectional relationship between anxiety disorders and these chronic illnesses [19]. SSRIs are generally preferred in such patients due to their favorable cardiovascular and metabolic safety profile, supporting the rationality of the prescribing pattern observed. Most patients (26 out of 40) did not report any adverse effects. Among those who did, dizziness, nausea and sedation were most common, with none requiring treatment discontinuation. According to Nerlekar *et al.* these findings are consistent with Indian observational studies reporting low incidence of serious adverse effects with SSRIs and judicious short-term benzodiazepine use [13]. Clinical outcomes were largely favorable, with an overall improvement rate of 77.5%. Patients who recovered were predominantly treated with SSRIs, either as monotherapy or combined with benzodiazepines, whose adjunctive use facilitated early symptom relief and better adherence. Patients without adequate improvement (22.5%) may represent cases with greater baseline severity, comorbid conditions, shorter follow-up, or adherence issues. The findings demonstrate that SSRIs formed the foundation of long-term management while benzodiazepines were judiciously used as short-term adjuncts, largely consistent with Indian Psychiatric Society guidelines advocating SSRIs and SNRIs as first-line agents [10, 18]. The favorable outcomes and low adverse drug reactions further support guideline-based pharmacotherapy in real-world practice. Overall, the present study highlights rational prescribing practices and satisfactory clinical outcomes in a tertiary care setting, underscoring the importance of individualized treatment, cautious benzodiazepine use and guideline-adherent pharmacotherapy. The relatively small sample size and single-centre design may limit generalizability; however, the prospective observational design provides useful real-world information regarding prescribing practices and clinical outcomes.

## Conclusion:

We report the prevailing pattern of first-line pharmacotherapy in newly diagnosed patients with anxiety and panic disorders. Selective serotonin reuptake inhibitors were the most commonly prescribed agents for long-term management, while benzodiazepines such as alprazolam and clonazepam were commonly used as short-term adjuncts for early symptom relief. The observed pattern reflects rational and guideline- oriented practice, with combination therapy being used as commonly as monotherapy in routine clinical practice.

## Advancement to knowledge: 

This study provides real-world evidence on first-line pharmacotherapy outcomes in newly diagnosed anxiety and panic disorder patients from a tertiary care setting in western India, highlighting rational, guideline-concordant prescribing practices with SSRIs as the pharmacological cornerstone and judicious short-term benzodiazepine use, contributing to the limited Indian observational literature on this subject.

## Figures and Tables

**Table 1:** Demographic and clinical characteristics of patients with anxiety disorder and panic disorder

**Parameter**	**Category**	**Number of Patients (%)**
Age (years)	Mean ± SD	36.3 ± 10.8
	Range	18 - 57
Age group	≤ 30 years	12 (30.0)
	31-45 years	17 (42.5)
	> 45 years	11 (27.5)
Sex	Male	21 (52.5)
	Female	19 (47.5)
Weight (kg)	Mean ± SD	65.7 ± 12.8
Diagnosis	Anxiety Disorder	29 (72.5)
	Panic Disorder	11 (27.5)
Comorbid conditions	None	27 (67.5)
	Hypertension	8 (20.0)
	Diabetes Mellitus	5 (12.5)
